# New Insights into the Anticancer Effects and Toxicogenomic Safety of Two β-Lapachone Derivatives

**DOI:** 10.3390/ph18060837

**Published:** 2025-06-03

**Authors:** José Rivaldo De Lima, Alexandre José Da Silva Góes, Elizabeth Fernanda De Oliveira Borba, Meykson Alexandre da Silva, Rodrigo Ribeiro Alves Caiana, Maria do Desterro Rodrigues, Mariza Severina De Lima Silva, Cristiano Aparecido Chagas, Blandine Baratte, Thomas Robert, Stéphane Bach, Isabelle Ourliac-Garnier, Pascal Marchand, Teresinha Gonçalves Da Silva

**Affiliations:** 1 Departamento de Antibióticos, Universidade Federal de Pernambuco, Recife 50670-420, Brazil; joserivaldo.lima@ufpe.br (J.R.D.L.); alexandre.goes@ufpe.br (A.J.D.S.G.); elizabethfernanda_7@hotmail.com (E.F.D.O.B.); rodrigo.caiana@ufpe.br (R.R.A.C.); maria.drodrigues@ufpe.br (M.d.D.R.); mariza.slima@ufpe.br (M.S.D.L.S.); 2 Centro Acadêmico de Vitória, Universidade Federal de Pernambuco, Vitória de Santo Antão 55608-680, Brazil; meykson.silva@ufpe.br (M.A.d.S.); cristiano.chagas@ufpe.br (C.A.C.); 3 Integrative Biology of Marine Models Laboratory (LBI2M), Station Biologique de Roscoff, Sorbonne Université, CNRS, UMR8227, F-29680 Roscoff, France; baratte@sb-roscoff.fr (B.B.); thomas.robert@sb-roscoff.fr (T.R.); bach@sb-roscoff.fr (S.B.); 4 Plateforme de Criblage KISSf (Kinase Inhibitor Specialized Screening Facility), Station Biologique de Roscoff, Sorbonne Université, CNRS, FR2424, F-29680 Roscoff, France; 5 Cibles et Médicaments des Infections et de L’immunité, IICiMed, UR 1155, Nantes Université, F-44000 Nantes, France; isabelle.ourliac@univ-nantes.fr (I.O.-G.); pascal.marchand@univ-nantes.fr (P.M.)

**Keywords:** quinone, thiosemicarbazone, apoptosis, necrosis, kinases, DNA damage

## Abstract

**Background/Objectives:** β-Lapachone (β-lap) is an *o*-naphthoquinone with potent antitumor activity. However, its clinical application is hindered by poor solubility and toxicity. Thiosemicarbazone derivatives of β-lap (BV3 and BV5) have demonstrated enhanced selectivity and anticancer efficacy in leukemia cells. Therefore, this study aimed to evaluate the therapeutic potential of these derivatives in solid tumors. Furthermore, the mechanism of tumor cell death, the involvement of protein kinase inhibition, and the toxicogenetic safety of BV3 and BV5 were investigated. **Methods**: The cytotoxic effects of BV3 and BV5 were assessed in cancer cell lines and a non-cancerous cell line. The compounds were most effective against HeLa (human cervical adenocarcinoma) cells. For that reason, this type of cell was chosen to study how the compounds might cause cell death, using flow cytometry. Kinase inhibition assays were conducted in vitro and in silico, followed by genotoxicity assessments to determine toxicogenetic safety. **Results**: BV3 and BV5 derivatives significantly inhibited cancer cell proliferation after 72 h, with IC_50_ values ranging from 2.8 to 36.9 µM. BV3 demonstrated superior selectivity (selectivity index: 15.6) when compared to β-lap (selectivity index: 1.9) in HeLa cells. Morphological changes and flow cytometry analysis revealed features of apoptosis and/or necrosis in HeLa cells treated with the compounds BV3 and BV5. Furthermore, among the kinases tested, BV3 and BV5 were more effective in inhibiting the activity of the protein kinases JAK3 and GSK3β. This result was also confirmed by the in silico studies. Additionally, genotoxicity assays indicated an overall favorable toxicogenetic safety profile; however, BV5 exhibited potential genotoxicity at high concentrations. **Conclusions**: The findings underscore the anticancer potential of BV3 and BV5 in solid tumors and highlight their mechanism of action, which involves protein kinases. The findings also show that the drugs are selective and relatively safe.

## 1. Introduction

β-Lapachone (β-lap) is an *o*-naphthoquinone extracted from the heartwood of the tree *Handroanthus impetiginosus* (Mart. ex DC.) Mattos (synonym *Tabebuia avellanedae*), commonly known in Brazil as “pau d’arco” or “ipê roxo” [1,2]. β-lap presents several activities described in the literature, such as anticancer, antimicrobial, antiviral, anti-inflammatory, antiobesity, antioxidant, and neuroprotective activities, among others [2].

According to data from Clinical Trials (https://clinicaltrials.gov), β-lap has been tested in various conditions, including advanced solid tumors, carcinomas, and lymphomas. The first chemical formulation of β-lap with hydroxypropyl-β-cyclodextrin (HPβCD), known as ARQ 501 (ArQule, Woburn, MA, USA), either alone or in combination with other antineoplastic agents, has progressed to phase II clinical research. However, it was observed that patients developed hemolytic anemia due to complexation with HPβCD. Additionally, clinical studies with ARQ 501 as a cell-cycle inhibitor targeting E2F were carried out [3,4,5]. Subsequently, the formulation ARQ 761 (ArQule, Burlington, MA, USA), which is more water-soluble and has a lower concentration of HPβCD, was tested clinically up to phase I in advanced and refractory solid tumors related to tumors with high levels of NADPH: quinone oxidoreductase 1 (NQO1) [6]. However, the clinical utility of biologically NQO1-activated drugs has been limited up to date [6]. In clinical trials, various effects of ARQ 761 were observed in patients, including anemia (79%), fatigue (45%), hypoxia (33%), methemoglobinemia (26%), nausea (17%), and vomiting (17%). The maximum tolerated dose was found to be 390 mg/m^2^ [6]. Therefore, despite its potential antitumor activity, β-lap exhibits high toxicity, which has encouraged the development and synthesis of new derivatives with greater selectivity and fewer adverse effects [7].

**Figure 1:**
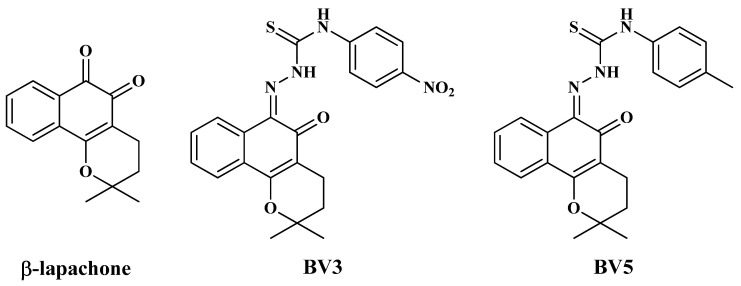
Chemical structures of β-lapachone, BV3, and BV5.

Recently, our research group showed that compounds derived from β-lap, 2-(2,2-dimethyl-5-oxo-3,4-dihydro-2H-benzo[h]chromen-6(5H)-ylidene)-N-(4nitrophen-yl)thio-carbohydrazide (BV3) and 2-(2,2-dimethyl-5-oxo-3,4-dihydro-2H-benzo[h]chromen-6(5H)-ylidene)-N-(p-tolyl)-thiocarbohydrazide (BV5) (Figure 1), presented anti-leukemic potential elucidated in vitro [7]. These compounds showed potent cytotoxic effects against the HL60 (human promyelocytic leukemia cells), K562 (human chronic myelogenous leukemia cells), K562-Lucena, and MOLT-4 (T lymphoblast cells), and low cytotoxicity against human peripheral blood mononuclear cells (PBMC), with BV3 standing out for its high selectivity [7]. Moreover, these derivatives were classified as Category 5 for toxicity according to the Globally Harmonized System of Classification and Labelling of Chemicals, as no deaths were observed in acute toxicity experiments at a dose of 2000 mg/kg [7]. However, currently, there is no available evidence regarding the effects of BV3 and BV5 on solid tumor cell lines, which contrasts with β-lap clinical focus on solid tumors.

The derivatives BV3 and BV5 differ from β-lap by their conjugation with thiosemicarbazone moiety. In this context, thiosemicarbazones are known to exhibit various biological activities, including anticancer, antiviral, and antibacterial activities, among others [8,9,10]. Molecules containing thiosemicarbazone can also act by inhibiting kinases important for cell survival and proliferation [11]. Furthermore, they represent a pharmacophore that is capable of effectively blocking the activity of ribonucleotide reductase (RNR), a critical enzyme that controls the rate of DNA synthesis in tumor cells [12]. They also inhibit Poly ADP ribose polymerase-1 (PARP-1), which plays an important role in DNA repair, gene transcription, and apoptosis in cancer cells [13].

Both β-lap and thiosemicarbazones are associated with genotoxic damage. Therefore, it is necessary to investigate DNA damage of new drugs with these nuclei [14,15,16]. Furthermore, in view of the lack of the studies about the potential of these derivatives in solid tumor cell lines and toxicogenetic safety, the aim of this work was to investigate the anticancer activity in solid tumors, mechanism of cell death, involvement of kinases in vitro and the toxicogenetic safety of BV3 and BV5 compounds. Finally, the affinity of the compounds for kinases JAK3 and GSK3β was evaluated in silico.

## 2. Results

### 2.1. Cellular Cytotoxicity

**Figure 2:**
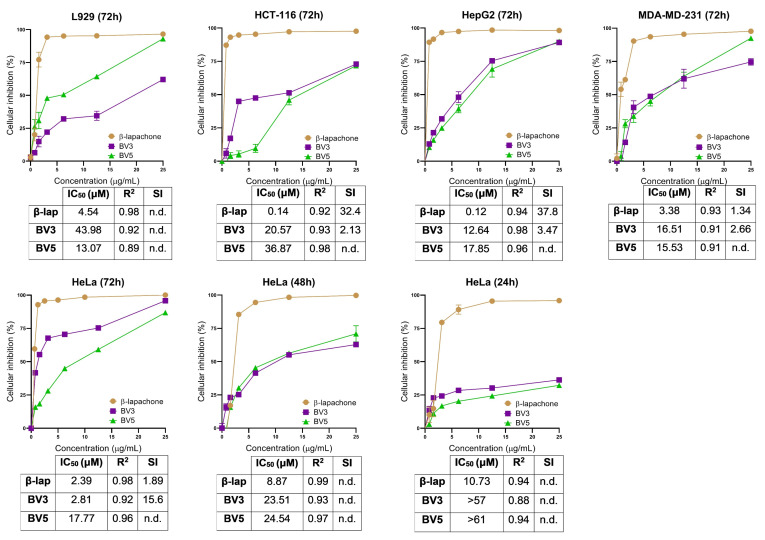
Cellular inhibition percentage of compounds BV3 and BV5 in the normal L929 cell line and the tumor cell lines HCT-116, HepG2, MDA-MD-231, and HeLa obtained through the MTT test. β-lap was used as a positive control. The data are presented as a mean and standard deviation. Below each graph, the IC_50_ (µM) of the compounds, R-squared (R^2^), and selectivity index (SI) are depicted accordingly. Not determined or <1 (n.d.). The experiment was performed in triplicate in two independent experiments (*n* = 6).

The cytotoxicity derived from BV3 and BV5 was assessed in HeLa (human cervical adenocarcinoma), HCT-116 (human colon carcinoma), HepG2 (human hepatocellular carcinoma), MDA-MB-231 (human breast adenocarcinoma), and non-cancerous L929 (murine fibroblasts), for 72 h of treatment (Figure 2).

The cytotoxicity of β-lap, BV3, and BV5 was first evaluated using the L-929 cell line, and IC_50_ values were 4.54, 43.98, and 13.07 µM, respectively. For cancer cells, β-lap exhibited IC_50_ values ranging from 0.12 to 3.38 µM. Meanwhile, BV3 displayed IC_50_ values ranging from 2.81 to 20.57 µM, and BV5 exhibited IC_50_ values between 15.53 and 36.87 µM. Due to the higher activity observed in the HeLa cell line, cytotoxicity was assessed at both 48 and 24 h time points. β-lap exhibited IC_50_ values of 8.87 µM and 10.73 µM for the 48 and 24 h, respectively, against HeLa. On the other hand, BV3 displayed IC_50_ values of 23.51 µM and >57 µM, and BV5 showed IC_50_ values of 24.54 µM and >61 µM for the 48 and 24 h, respectively.

The results indicated that compound BV3 exhibited a selectivity of 15.6 for the HeLa cell line, which was eight times higher than that of its precursor compound (β-lap) for the same cell line. Therefore, based on these data, the HeLa cell line was chosen to proceed with the tests due to its IC_50_ and IS results. Consequently, the IC_50_ values were investigated at 24 h and 48 h for the mechanism of action tests. β-lap was used as a standard, displaying IC_50_ values of 10.73 and 8.87 µM at incubation times of 24 h and 48 h, respectively. β-lap was used as a standard because, in addition to being a precursor to the compounds under study, it is a substance that has made it to the clinical trial stage.

### 2.2. Morphological Alterations in HeLa

**Figure 3:**
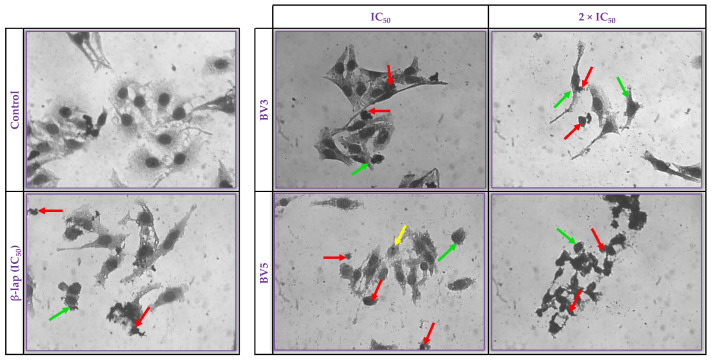
Morphological analysis of HeLa cells after 72 h of treatment with BV3 and BV5 at IC_50_ and 2 × IC_50_ concentrations were conducted using rapid panoptic staining and visualization through optical microscopy. The control group (Control) cells were treated with the vehicle (DMSO 0.1%). β-lap served as a standard at 2.4 µM. Staining with rapid panoptic allowed visualization under an optical microscope at a 400× magnification. Arrows indicate observed features: cellular volume reduction (green arrow), debris (red arrow), and pyknotic nucleus (yellow arrow). The experiment was performed in triplicate in two independent experiments (*n* = 6).

Cell morphology was examined by light microscopy after treatment of HeLa cells with BV3, BV5, or β-lap for 72 h to follow the late stage of cell death (Figure 3). Untreated HeLa cells (control) exhibited the typical morphology of adherent cells with intact nuclei, normal cell membrane, pleomorphic cells, and the presence of cells in mitosis. Cells treated with BV3 at concentrations of IC_50_ (2.81 µM) and 2 × IC_50_ (5.62 µM) displayed a reduced cellular volume, cells with condensed chromatin, and cellular debris. Cells treated with BV5 at concentrations of IC_50_ (17.77 µM) and 2 × IC_50_ (35.54 µM) showed morphological changes such as a loss of membrane integrity, reduced cellular volume, presence of fragmented/pyknotic nuclei, chromatin condensation, and cellular debris. Also, morphological alterations were concentration-dependent on cells treated with BV3 and BV5.

### 2.3. Flow Cytometry Analysis

**Figure 4:**
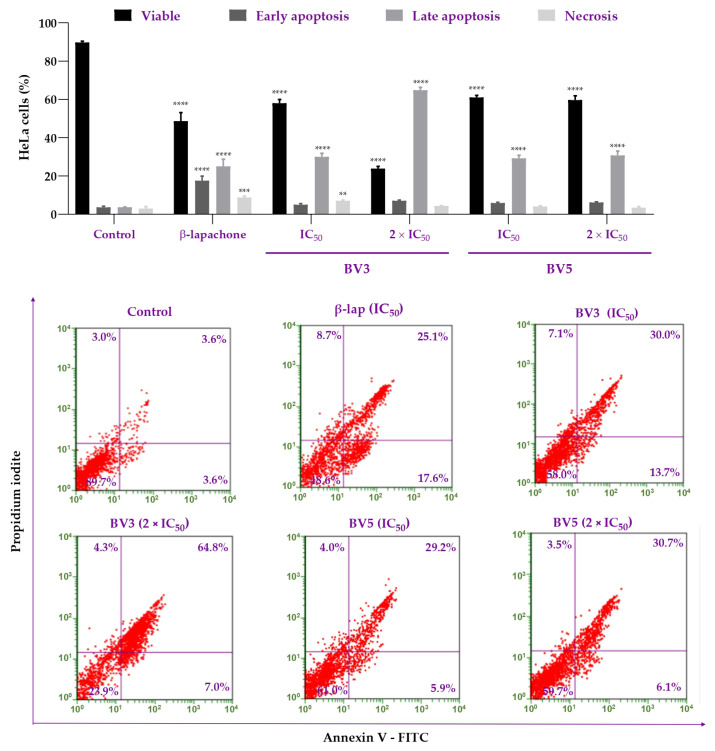
Effects of BV3 and BV5 compounds on HeLa cells after 48 h of incubation with the compounds. Cells were incubated with concentrations corresponding to the IC_50_ and 2 × IC_50_ of BV3 (23.5 and 47.0 µM), and BV5 (24.54 and 49.08 µM) obtained at the 48 h time point. β-lap was used as a positive control (8.87 µM) labeled with Annexin V-FITC and propidium iodide. Data are expressed as mean ± SEM. Statistical significance was determined by comparing the treatment to the negative control using ANOVA followed by Dunnett’s test, with *p* < 0.05 considered significant. (****) *p* < 0.0001 (***) *p* = 0.0001 (**) *p* = 0.0019. The experiment was performed in triplicate in two independent experiments (*n* = 6).

To confirm the cytotoxic activity of BV3 and BV5 compounds in the HeLa cells, cell death through apoptosis and/or necrosis was investigated using flow cytometry (Figure 4). For this, an intermediate time of 48 h was chosen to map the cell death pathway and not only the final stage of death.

Our results demonstrated that BV3 and BV5 compounds significantly decreased cell viability at both concentrations, IC_50_ or 2 × IC_50_ (*p* > 0.0001). Similarly, β-lap reduced the number of viable cells compared to the negative control (*p* > 0.0001). These results highlight that the compounds exhibit cytotoxic activity against the HeLa cell line. Regarding early apoptosis, no difference was observed between the groups treated with the derivatives and the negative control (*p* > 0.05). Only β-lap significantly increased the number of cells in early apoptosis (*p* > 0.0001). However, concerning late apoptosis, an increase in the number of marked cells was observed in all treated groups (*p* > 0.0001). Furthermore, regarding necrosis, increases were only noted in the BV3 treatment at the IC_50_ concentration (*p* = 0.0019) and β-lap (*p* = 0.0001).

### 2.4. In Vitro and In Silico Analyses of Kinase Inhibition

An in vitro functional assay was utilized to evaluate the efficacy of BV3 and BV5 against a panel of eight distinct human protein kinases implicated in a variety of human pathologies, including cancer [17,18]. BV3 and BV5 displayed kinase inhibitory activity at concentration of 10 µM for cyclin-dependent kinase 5 (CDK5/p25), cyclin-dependent kinase 9 (CDK9/CyclinT), Provirus Integration site for Moloney Leukemia Virus 1 (PIM1), glycogen synthase kinase 3β (GSK3β), casein kinase 1ε (CK1ε), Abelson tyrosine-protein kinase 1 (ABL1) and Janus kinase 3 (JAK3). Similarly, β-lap tested at 10 µM, showed a slight inhibition of the same target (Table 1).

**Figure 5:**
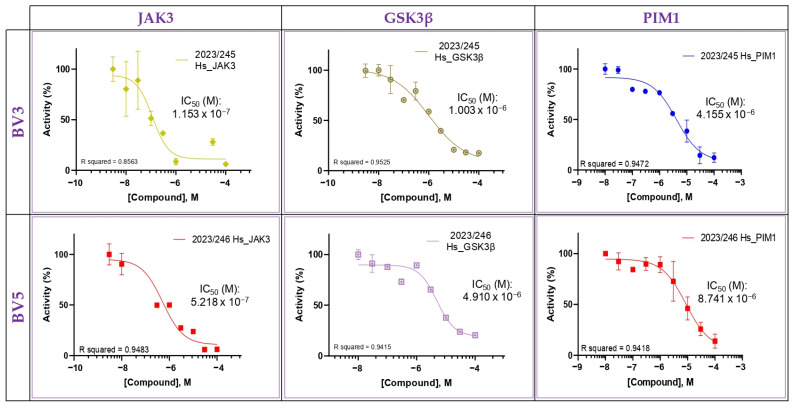
Kinase inhibition assay. Determination of IC_50_ in dose–response curves on GSK3β, PMI1, and JAK3. The data are presented as a mean and standard deviation. The experiment was performed in duplicate in four independent experiments (*n* = 8).

Furthermore, the IC_50_ for the most sensitive targets were determined. Thus, the IC_50_ values obtained for JAK3 were 0.11 µM and 0.52 µM; for GSK3β 1.00 µM and 4.91 µM; and for PIM1 4.15 µM and 8.74 µM for BV3 and BV5, respectively (Figure 5).

Then, to better understand this interaction, BV3 and BV5 compounds were subjected to molecular docking studies in order to evaluate their potential to anchor at the active site of the most sensitive kinases in the in vitro evaluation (JAK3 and GSK3β). The redocking step with the ligands co-crystallized in the selected structures (ML1 for JAK3 and 679 for GSK3β) demonstrated the adequacy of the parameters chosen for the molecular docking studies, obtaining RMSDs of 0.33 Å and 1.40 Å, respectively, and satisfying the standards required by the literature [19,20].

Similar to the behavior observed in the in vitro evaluation, compounds BV3 and BV5 demonstrated strong in silico affinity for JAK3 and GSK3β (Table 2). The most favorable values of free binding energies were obtained when compounds BV3 and BV5 anchored to the JAK3 structure (−10.25 kcal/mol and −10.08 kcal/mol, respectively), generating a more favorable interaction than that observed for the co-crystallized ligand of that structure (ML1, −8.99 kcal/mol). It is worth mentioning that good free binding energies were also observed when the compounds were docked inside GSK3β, exhibiting free binding energies values close to those found for their co-crystallized ligand (679).

**Figure 6:**
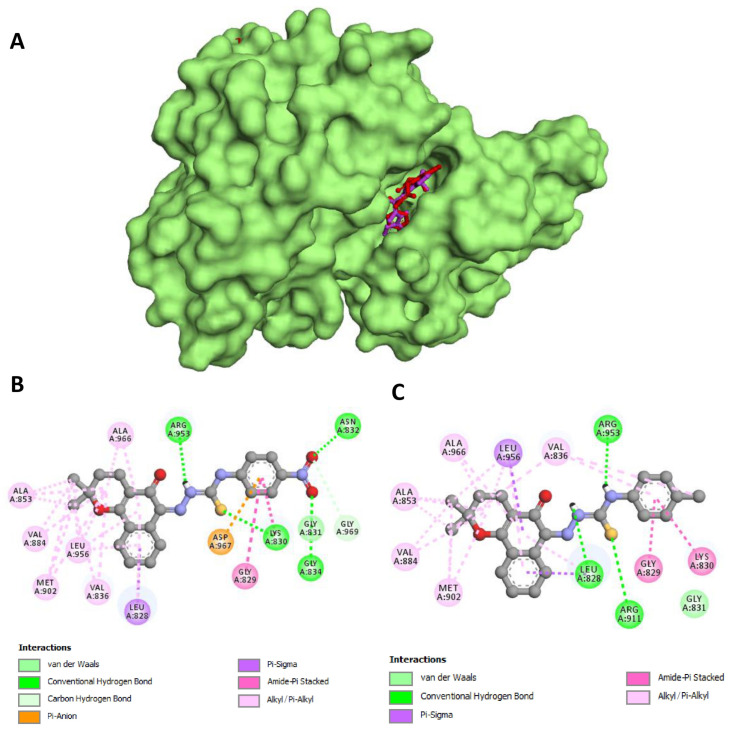
Detailed view of the docking conformations for compound BV3 and BV5 in the active site of JAK3 (PDB: 3LXK). Lower energetic conformation of BV3 (violet) and BV5 (orange) in the kinase domain of JAK3 (**A**). It is also possible to notice the 2D interactions’ maps with JAK3 amino acids of BV3 (**B**) and BV5 (**C**).

In addition, BV3 and BV5 were able to satisfactorily mimic the amino acid interactions formed between ML1 and 679 inhibitors with the catalytic domains of these enzymes (Table 2 and Figure 5 and Figure 6). It is also interesting to note that both compounds demonstrated greater affinity for the kinases than their synthetic precursor (β-lap), highlighting the value of the synthetic modifications performed to obtain the compounds BV3 and BV5.

As shown in Figure 6B, the molecular docking analysis showed that the naphthoquinone moiety of compound BV3 formed important hydrophobic interactions with the catalytic site of JAK3, establishing alkyl and pi–alkyl interactions with Leu828, Val836, Ala853, Val884, Met902, Leu956, and Ala966. The pi electron cloud of the lateral benzene ring established pi–anion interactions with Asp967 and amide–pi stacked with Gly829 and Lys830. In turn, the thiosemicarbazone moiety established strong hydrogen bonds with Lys830 and Arg953, while the nitro group was able to form hydrogen bonds with Asn832 and Gly834 and carbon hydrogen with Gly831 and Gly969.

Similar behavior was observed for compound BV5 (Figure 6C), in which the interactions of the naphthoquinone moiety were formed with the same amino acids that interacted with this moiety of compound BV3. The exchange of the nitro group made the side chain more hydrophobic, providing Van der Waals interactions with Gly831, in addition to the amide–pi stacked interactions with Gly829 and Lys830. Hydrogen bonds were again observed for the thiosemicarbazone moiety, formed with Leu828, Arg911, and Arg953.

**Figure 7:**
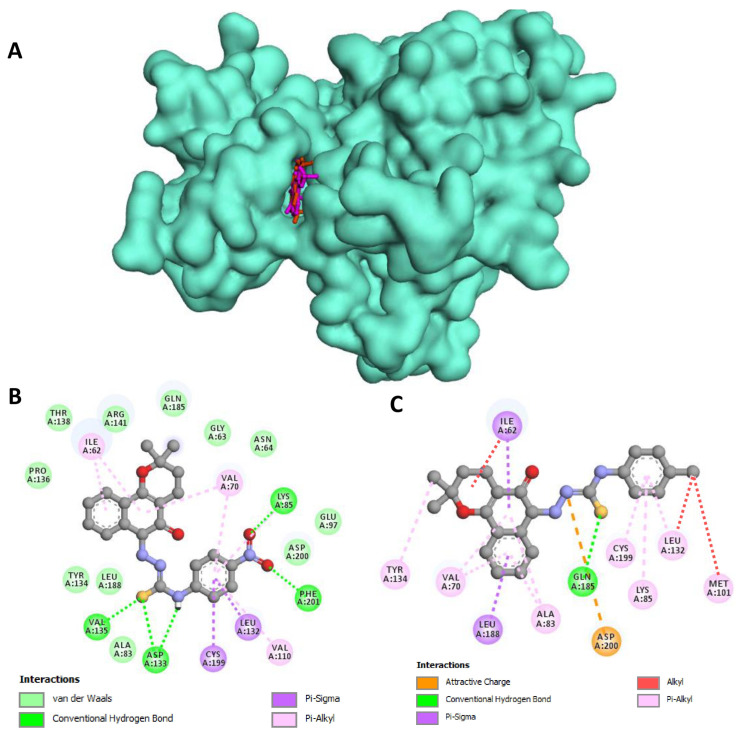
Detailed view of the docking conformations for compound BV3 and BV5 in the active site of GSK3β (PDB: 1Q4L). Lower energetic conformation of BV3 (violet) and BV5 (orange) in the active site of GSK3β (**A**). It is also possible to note the 2D interactions’ maps with GSK3β amino acids of BV3 (**B**) and BV5 (**C**).

As in the in vitro evaluation, BV3 and BV5 also showed affinity for the active site of GSK3β (Figure 7A). As shown in Figure 7B, BV3 presented a good fit in the hydrophobic portion of the active site, forming Van de Waals interactions with Gly63, Asn64, Ala83, Glu97, Tyr134, Pro136, Thr138, Arg141, Gln185, Leu188, and Asp200, in addition to pi–alkyl and pi–sigma interactions with Ile62, Val70, Leu132 and Cys199. Additionally, hydrogen bonds were formed between the thiosemicarbazone moiety with Asp133 and Val135, and between the nitro group with Lys85 and Phe201.

A significant number of hydrophobic interactions were also observed in the docking of the compound BV5 on GSK3β protein (Figure 7C), especially of the alkyl, pi–alkyl and pi–sigma type with Ile62, Val70, Ala83, Lys85, Met101, Leu132, Tyr134, Leu188, and Cys199. In addition, the sulfur of the thiosemicarbazone moiety was able to form hydrogen bonds with Gln185, and an attractive charge interaction was also observed between Asp200 and one of the thiosemicarbazone nitrogens.

### 2.5. Toxicogenomic Safety

**Figure 8:**
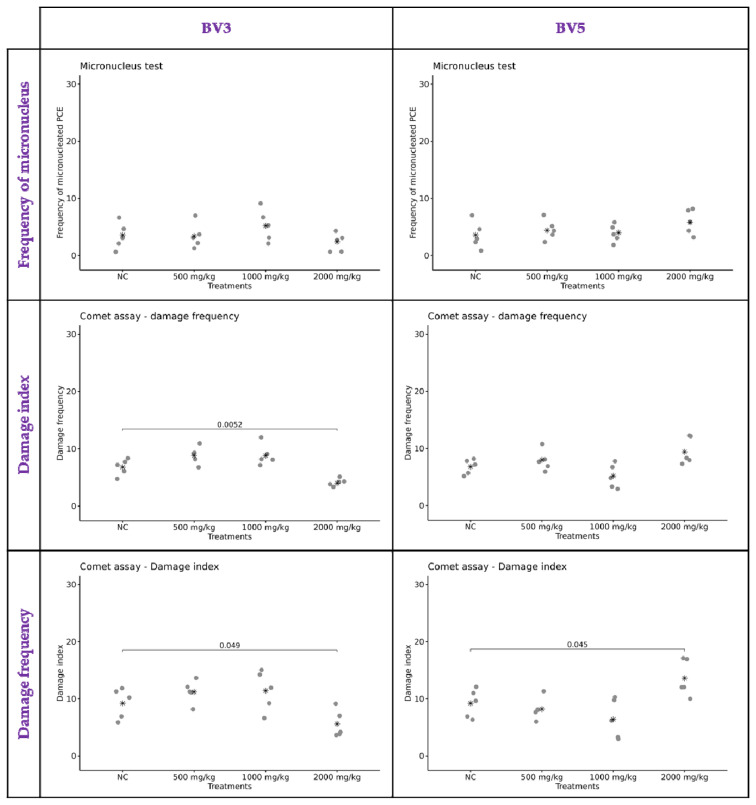
Detection of genotoxic and mutagenic effects through the parameters of micronucleus frequency (PCE), damage index (DI) and damage frequency (DF). The negative control treated with vehicle (NC) and those treated with BV3 and BV5 at doses of 500, 1000, and 2000 mg/kg were subjected to a comet assay and micronucleus test, using peripheral blood erythrocytes from *Swiss* mice. The mean for each group is indicated by an asterisk. A significative difference is observed in all comparisons when *p* < 0.05 (*n* = 5).

Furthermore, considering the anticancer properties of the derivatives, biological safety parameters were also examined through their genotoxic and mutagenic potential. Compounds BV3 and BV5 were tested at three doses (500, 1000, and 2000 mg/kg). The results are presented in Figure 8. Compared to the negative control group, no differences in the number of micronuclei were observed for BV3. The doses of 500 and 1000 mg/kg showed no significant differences in the damage index or damage frequency obtained from the comet assay. However, it was noted that at a dose of 2000 mg/kg, there was a decrease in the basal damage index and frequency (*p* = 0.0052 and 0.049, respectively). The groups treated with BV5 at different doses showed no differences in the number of micronuclei compared to the negative control. The doses of 500 and 1000 mg/kg exhibited no differences in the damage index; however, an increase was observed in the group treated with 2000 mg/kg of BV5 (*p* = 0.045). Thus, BV5 treatment at 2000 mg/kg resulted in an elevated damage index in the comet assay, indicating genotoxicity only at the highest concentration. However, this damage can be repairable and may not induce mutagenicity.

## 3. Discussion

### 3.1. Cellular Cytotoxic

In this study, we sought to investigate the cytotoxic action of the compounds BV3 and BV5 on solid tumor cells, since the action of these compounds had only been tested in leukemia. Thus, among the cells tested (HeLa, HCT-116, HepG2, and MDA-MB-231), BV3 and BV5 showed a promising activity against HeLa, with a high level of cytotoxicity and selectivity. Our hypothesis was that this selectivity would be explained by the high level of NQO1 in female genitourinary tumors, as this enzyme is strongly associated with the progression of these tumor types, as indicated by previous studies [21,22]. Moreover, an investigation with BV3 and BV5 showed that co-treatment with dicoumarol, an NQO1 inhibitor, resulted in reduced cytotoxicity of these compounds [7]. Additionally, it is known that β-lap undergoes redox cycles leading to an increase in reactive oxygen species (ROS) levels within cells, with NQO1 playing a crucial role in this process [7,23,24]. Then, BV3 and BV5 may still share similar mechanisms with their precursor, β-lap. In addition, as BV3 and BV5 contain a thiosemicarbazone moiety, they confirm findings in the literature that thiosemicarbazone derivatives can induce selective cytotoxicity in HeLa cells [25,26]. Also, derivatives of thiosemicarbazones can induce cytotoxicity and act through the induction of apoptosis by DNA binding activity and the upregulation of caspase −3, −8, and −9 in HeLa cells [27]. Thus, BV3 and BV5 may still have similar mechanisms of action to β-lap but with better selectivity for HeLa cells due to the thiosemicarbazone moiety.

### 3.2. BV3 and BV5 Increased Apoptosis in HeLa Cells

A morphological analysis was performed to observe cellular changes indicative of cell death by apoptosis or necrosis. Changes in cell morphology suggested apoptotic cell death in HeLa cells treated with BV3 and BV5 derivatives. Similar changes were observed in cells treated with β-lap. According to the literature, β-lap can act through various cell death pathways, such as apoptosis, necrosis, autophagy, and ferroptosis in cancer cells [7,28]. Also, thiosemicarbazone derivatives can induce apoptosis in HeLa cells [27]. However, in leukemia cell lines (non-adherent cells), BV3 and BV5 molecules mainly induce autophagy cell death [7]. This interesting result shows that BV3 and BV5 can act differently depending on the cellular context.

Thus, to confirm the capacity of BV3 and BV5 to induce apoptosis in HeLa cells, we performed a flow cytometry analysis. The results showed that BV3 and BV5 could induce apoptosis in IC_50_ and 2 × IC_50_ concentrations. The compound BV3 exhibited a dose–response effect in late apoptosis. At the IC_50_ concentration, an increase in the percentage of necrosis was observed. This supports data from the literature, which indicate that β-lap can activate the necrosis pathway due to DNA damage in cancer cells [29,30]. Also, β-lap activates the apoptotic death pathway in breast cancer, colon cancer cell lines [5], and myeloma cells [31].

Furthermore, it is important to emphasize that the activity of compounds BV3 and BV5 interferes with the inhibition of NQO1 [7]. The literature has already pointed out that the β-lap mechanism of apoptosis induction involves NQO1 [32,33,34]. Thus, due to high NQO1 levels in HeLa cells [21,22], apoptosis is expected, and it was confirmed in Andrade et al. [7] and in our findings.

The BV3 and BV5 compounds act differently than the precursor, β-lap, due to the thiosemicarbazone moiety. It has been observed that BV3 and BV5 are more selective, less cytotoxic, and induce apoptosis/necrosis in HeLa cells. Then, other thiosemicarbazone derivatives can act through the induction of apoptosis; for example, the compound di-2-pyridylketone 4,4-dimethyl-3-thiosemicarbazone (Dp44mT) with a thiosemicarbazone moiety induces apoptosis through the downregulation of Interleukin-6 (IL-6) and Janus Kinase 2/Signal Transducer and Activator of Transcription 3 (JAK2/STAT3) pathway in glioma [35]. However, the compound Dp44mT also showed that it could cause both autophagy and apoptosis in pancreatic cancer cells due to the activation of the 5′-adenosine monophosphate-activated protein kinase (AMPK) pathway [36]. Thus, this work corroborates the hypothesis that thiosemicarbazones and/or lapachone derivatives can cause different types of cell death.

### 3.3. BV3 and BV5 Can Inhibit Kinases

Kinases are known to play an important role in the initiation, progression, and survival of cancer cells. The human genome encodes 538 protein kinases, and new kinase inhibitors are important pharmacological targets in cancer therapy [37]. Therefore, we tested the hypothesis that β-lap and BV3 and BV5 could interfere with the action of CDKs, GSK3β, and other kinases for cancer therapy. Then, in our findings, we observed that 10 µM of BV3 and BV5 could affect the functional activity of CDK5/p25, CDK9/CyclinT, PIM1, GSK3β, ABL1, and JAK3. However, the compounds showed prominent inhibition of JAK3 and GSK3β.

JAK3 is a cytoplasmic non-receptor tyrosine kinase that plays a role in cytokine pathways and growth factor receptors, and its mutation is frequently described in cancers such as lymphomas and leukemias [38]. Therefore, JAK3 inhibition has a favorable effect on antineoplastic therapy, with studies showing that irreversible JAK3 inhibitors form a covalent bond with the Cys909 residue of JAK3 [39,40]. In our in silico studies, BV3, BV5 and β-lap showed high affinity for the ATP-binding cavity of JAK3 (low free binding energies), although it did not form covalent interactions with Cys909, supporting the hypothesis that these compounds function as reversible JAK3 inhibitors. The inhibition by BV3 and BV5 can be attributed to the thiosemicarbazone moiety, since the compounds showed greater inhibition than the precursor β-lap.

GSK3β is an important kinase whose dysregulation promotes tumor resistance, evasion of cell death, and proliferation and invasion of cancer cells [40]. Also, it is known that the inhibition of GSK3β has a therapeutic effect on 25 types of cancer [40]. Additionally, β-lap was shown to inhibit GSK3β in mouse brain tissue [41]; therefore, the inhibition of this target was expected. Then, our results showed that the both BV3 and BV5 could inhibit GSK3β, and moreover, the level of inhibition was higher compared to β-lap.

Thus, these results reinforce the hypothesis that the anticancer activity of BV3 and BV5 is related to the inhibition of kinases with roles in tumor survival and progression. These results will pave the way for the further development of new β-lap derivatives as pharmaceutical drugs affecting kinases for cancer target therapy.

### 3.4. BV3 and BV5 Have Toxicogenetic Safety

In this study, we also investigated the toxicogenetic safety of BV3 and BV5. This preclinical assay was important to make sure that these compounds did not represent a risk for DNA damage in non-cancer cells. In our results, BV3 did not reveal genotoxic effects on peripheral blood mononuclear cells from mice. Interestingly, it was noted that BV3 could decrease the basal damage index and frequency of DNA; this suggests effective mechanisms for DNA repair and the reduction in genomic instability, implying improved cellular viability and lower risk of mutations. However, new studies to better understand the capacity of these compounds to be antimutagenic is imperative to affirm this. On the other hand, it is known that β-lap can offer protective effects due to its activation mechanism mediated by the NQO1 of antioxidant enzymes in non-neoplastic cells and tissues [42,43,44]. These results corroborate the evidence that β-lap derivatives can act as protectors for cellular stresses in non-cancer cells.

BV5 treatments showed no difference in frequency of damage or micronuclei at any of the tested doses. However, BV5 increased the damage index at the highest dose of 2000 mg/kg. Therefore, BV5 can be genotoxic but not mutagenic at 2000 mg/kg. The literature reveals that derivatives of β-lap, such as Nor-β-lapachone, do not induce DNA breakage or chromosomal aberrations in non-cancerous cells at low concentrations. However, at high concentrations in vitro, Nor-β-lapachone can induce oxidative modifications of DNA bases indirectly through the generation of ROSs, but not through DNA intercalation [15]. In addition, thiosemicarbazone compounds is known as an inhibitor of enzymes involved in DNA synthesis, DNA repair, and gene transcription [12,13]. It is also worth noting that the inhibition of healthy cell growth (L929—Figure 2) and the increase in the damage index observed for BV5 may be associated with the presence of the nitro group, which is sometimes referred to in the literature as a toxicophore capable of inducing mutagenicity/genotoxicity. However, its exclusively toxic role is still debated in medicinal chemistry, as reports have highlighted its involvement in the formation of anticancer, antitubercular, and antiparasitic pharmacophores [45].

In summary, BV3 and BV5 exhibited no mutagenic activity at the tested doses, as confirmed by micronucleus test results. Thus, the compounds demonstrated toxicogenetic safety. However, caution is advised regarding BV5’s genotoxicity at high doses. Further in vivo dose-optimization studies are encouraged to better understand the safety of these compounds, particularly BV5, which exhibited genotoxicity at 2000 mg/kg.

## 4. Materials and Methods

### 4.1. Reagents

Dulbecco’s Modified Eagle’s Medium (DMEM), Roswell Park Memorial Institute 1640 Medium (RPMI-1640), penicillin/streptomycin (10,000 U/mL), and Fetal Bovine Serum (FBS) were purchased from Gibco™, Waltham, MA, USA); 3-(4,5-dimethylthiazol-2-yl)-2,5-diphenyltetrazolium bromide (MTT; Trocris Bioscience, Bristol, UK); Annexin V Apoptosis Detection Kit (eBioscience™, San Diego, CA, USA); ketamine and xylazine (Ceva, São Paulo, Brazil); dimethyl sulfoxide (DMSO, Neon, Brazil); acridine orange, low-melting-point agarose, standard agarose and tris(hydroxymethyl)-aminomethane (Sigma-Aldrich, St. Louis, MO, USA); Triton X-100 (Neon, Brazil). EDTA P.A., NaCl P.A., NaOH P.A., and HCl P.A. have been acquired from Dinâmica^®^, São Paulo, Brazil.

### 4.2. Molecules

The molecules tested are two semi-synthetic compounds derived from β-lap, 2-(2,2-dimethyl-5-oxo-3,4-dihydro-2H-benzo[h]chromo-6(5H)-ylidene)-N-(4-nitrophenyl)hydrazinecarbo-thioamide (BV3) and 2-(2,2-dimethyl-5-oxo-3,4-dihydro-2H-benzo-[h]chromo-6(5H)-ylidene)-N-(p-toluil)-hydrazinecarbothioamide (BV5). The synthesis process was previously described by de Andrade et al. [7].

### 4.3. Cell Lines

Solid tumors cell lines HeLa, HCT-116, HepG2, MDA-MB-231, and non-cancerous cell line L929 were used. The cells were obtained from the Cell Bank of Rio de Janeiro, (BCRJ) Rio de Janeiro, Brazil. HeLa and L929 cells were cultured in flasks using Dulbecco’s Modified Eagle’s Medium, pH 7.2–7.4, supplemented with 1% penicillin/streptomycin (10,000 U/mL) and 10% FBS. On the other hand, HCT-116, HepG2, and MDA-MB-231 cell lines were maintained in Roswell Park Memorial Institute 1640 Medium (RPMI 1640), pH 7.2–7.4, supplemented with 1% penicillin/streptomycin and 5% FBS. Cells were monitored under an optical inverted microscope (XD-202, Nexcope™, Ningbo, China) and kept incubated at 37 °C in an atmosphere with 5% of CO_2_.

### 4.4. Cell Viability and Determination of IC_50_

To assess the cytotoxicity of the compounds, the MTT [3-(4,5-dimethylthiazol-2-yl)-2,5-diphenyltetrazolium bromide] assay was employed [46]. The cells (2 × 10^5^/mL) were incubated with the substances at concentrations ranging from 0.78 to 25 µg/mL. β-lap was used as a positive control. After 24, 48, or 72 h of incubation, MTT was added at a concentration of 5 mg/mL. Cell viability in different treatments was compared to the negative control using the following formula:

$$Cell\;viability\left(\%\right)=\left(\frac{mean\;absorbance\;of\;treated\;samples}{mean\;absorbance\;of\;negative\;control}\right)\times 100$$

The IC_50_ (the concentration that inhibits 50% of cell growth compared to the negative control) was calculated using nonlinear regression in GraphPad Prism 8.0 for Windows (GraphPad Demo Software, San Diego, CA, USA). Experiments were performed in triplicate and in two independent experiments. The protocol is also described in other previous publications [7,47].

### 4.5. Selectivity Index (SI)

The IC_50_ of normal and cancer cells was used to calculate the SI of compounds BV3 and BV5. The SI indicated the selectivity of the compounds among the normal cell line (L-929) and a cancer cell line (HCT-116, MDA-MB-231, HepG2, or HeLa). SI is a crucial parameter for therapeutic applications in upcoming clinical trials [48]. Therefore, the following formula was used to calculate SI:

$$SI=\frac{IC50(normal\;cell\;line)}{IC50(cancer\;cell\;line)}$$

### 4.6. Morphological Analyses

Following the screening of IC_50_ values and SI in solid tumor cells, indications of potential cell death were explored in the most sensitive cell line. The morphological changes were evaluated according to protocols previously published with modifications [7]. Thus, HeLa cells were seeded in 12-well plates at a concentration of 4 × 10^5^ cells/mL and incubated overnight for adhesion. Subsequently, the cells were treated with substances for 72 h at the IC_50_ concentrations or 2 × IC_50_ concentrations. β-lap was used as a positive control and the culture medium as the negative control. After the treatment period, the medium was aspirated, and the plate was washed twice with PBS. The cells were fixed and stained using the Panoptic Rapid Kit following the manufacturer’s protocol. Morphological analyses were conducted to observe cellular alterations indicative of cell death, including cellular volume reduction, membrane integrity, karyolysis, pyknosis, karyorrhexis, apoptotic bodies, vacuoles, and cellular debris.

### 4.7. Evaluation of the Induction of Apoptosis or Necrosis

The analysis was performed using flow cytometry with the Annexin V Apoptosis Detection Kit (eBioscience™, USA) following the manufacturer’s instructions. Initially, HeLa cells were incubated at a concentration of 4 × 10^5^ cells/mL and treated with the compounds at their IC_50_ or 2 × IC_50_ values obtained at 48 h. β-lap was used as a positive control, and the culture medium as the negative control. After 48 h of treatment, the cells were trypsinized and centrifuged at 1200× *g* for 5 min. Subsequently, the cells were washed with PBS and centrifuged again. The cells were then resuspended in buffer and incubated with 5 µL of Annexin V-FITC for 10 min. Afterward, the cells were washed with the buffer, centrifuged, resuspended in the buffer, and stained with 10 µL of propidium iodide. The cells were acquired using the Guava EasyCyteHT cytometer (Merck-Millipore, Burlington, MA, USA) with Guava—Soft™ software version 2.7. A total of 5000 events were counted per treatment.

### 4.8. Protein Kinase Assays

Kinase enzymatic activities of CDK5/p25, CDK9/CyclinT, HASPIN, PIM1, GSK3β, CK1ε, ABL1, and JAK3 were assayed using the luminescent ADP detection assay described in Zegzouti et al. [49] (ADP-GloTM assay kit provided by Promega, Madison, WI, USA). Briefly, the reactions were carried out in appropriate kinase buffer, with either protein or peptide as substrate in the presence of 10 µM ATP. Assay conditions used for each kinase tested here are described in Ibrahim et al. [50]. The Envision (PerkinElmer, Waltham, MA, USA) microplate luminometer was used to measure the transmitted signal expressed in Relative Light Unit (RLU). In order to determine the half maximal inhibitory concentration (IC_50_), the assays were performed in duplicate in the absence or presence of increasing doses of the tested compounds. Kinase activities are expressed in % of maximal activity, i.e., measured in the absence of inhibitor but with a similar dose of DMSO (solvent of the tested compounds).

### 4.9. Molecular Docking

The binding potential of BV3 and BV5 to kinases was assessed through an in silico analysis. Thus, the three-dimensional structures of the ligands were built using the Avogadro 1.2.0 software [51] and optimized by the semi-empirical method PM6 [52] implemented in MOPAC2016. The ligands were transformed into “.pdb” format and prepared for docking by fusing the non-polar hydrogens, calculating the partial charge by the Gasteiger method and defining the rotatable bonds from the AutoDockTools-1.5.6 package. The three-dimensional structure of JAK3 (3LXK) and GSK3β (1Q4L) were obtained from the RCSB Protein Data Bank in “.pdb” format and prepared by removing water residues and other heteroatoms, fusing non-polar hydrogens and adding polar hydrogens and Kollman charges. The grid box was defined by taking as the central point the co-crystallized inhibitors of each structure, reaching the amino acids belonging to the binding site, choosing for JAK3 (ML1): x = 2.672; y = 15.107; z = 4.944, with dimensions of 40 × 30 × 28 points, and for GSK3β (679): x = 39.439; y = 6.675; z = 34.204, with dimensions of 30 × 38 × 36 points. Each ligand was subjected to a total of 100 runs of the Lamarckian genetic algorithm.

The listed parameters were validated through a redocking study involving co-crystallized inhibitors of each structure (ML1 for JAK3 and 679 for GSK3β), considering adequate the parameters capable of reproducing, in silico, the co-crystallized conformation with a Root-Mean Standard Deviation (RMSD) value ≤ 2 Å [19,20]. The resulting coupling conformations, as well as images showing enzyme-ligand interactions, were generated and analyzed using AutoDockTools, the BIOVIA Discovery Studio Visualizer.

### 4.10. In Vivo Assessment of Toxicogenetic Safety

The toxigenic safety of BV3 and BV5 in vivo was assessed in accordance with previously established methodology [53,54].

#### 4.10.1. Groups and Administration Protocol

Forty female *Mus musculus* mice (*Swiss* strain) were used. The animals were divided into 8 groups, with 5 animals each: G1—negative control (NC) treated with the vehicle solution intraperitoneally; G2—positive control (PC) treated intraperitoneally with cyclophosphamide at a dose of 30 mg/kg; groups exposed to the BV3 derivative—Group BV3—I intraperitoneally at doses of 2000 mg/kg; Group BV3—II 1000 mg/kg; Group BV3—III 500 mg/kg; and groups exposed to BV5—Group BV5—I, received BV5 intraperitoneally at doses of 2000 mg/kg; Group BV5—II 1000 mg/kg; and Group BV5—III 500 mg/kg. For the comet assay, approximately 0.5 mL of blood from each animal was collected via the tail vein 6–8 h after administration of the compounds. For the micronucleus test, 1 mL of blood from each animal was collected by cardiac puncture 48 h after administration of the compounds. For that collection, the mice were anesthetized with ketamine (100 mg/kg) and xylazine (10 mg/kg), intraperitoneally. Immediately after blood collection, the animals were euthanized with high doses of anesthetics (ketamine 300 mg/kg and xylazine 30 mg/kg). The in vivo experiments were approved by the Ethics Committee on the Use of Animals (CEUA) of the Federal University of Pernambuco, under process no. 004/2023.

#### 4.10.2. Alkaline Comet Assay

The female mice (*n* = 40) were equally distributed into 8 experimental groups; the groups were composed of 5 female animals each. After administering the solutions and compounds to the groups, blood samples were taken 6 and 8 h after treatment. Subsequently, 15 µL of blood was used, which was homogenized with 100 µL of low-melting-point agarose (LM agarose) and subsequently deposited on slides previously prepared with standard agarose. The slides were covered with coverslips and left at 4 °C for 10 min. After refrigeration, the coverslips were removed and the slides were placed in a lysis solution (2.5 M NaCl, 100 mM EDTA, 10 mM tris, 1% Triton x-100, 10% DMSO, pH 13) for 48 h. After lysis, the slides were left in an alkaline electrophoresis buffer (1 M NaOH and 200 mM EDTA disodium salt, pH 13) for 20 min to unroll the DNA and then subjected to electrophoresis in a horizontal vat for 20 min, with a current of ± 300 mA and potential difference of 32 V. After electrophoresis, the slides were neutralized for 15 min in 0.4 M tris-HCl buffer, pH 7.5, and fixed for 5 min in absolute ethanol. The slides were stained with 100 µL of a 1 µg/mL acridine orange solution. All test steps were carried out under red light. To analyze damage levels, 100 nucleoids from each animal were evaluated for damage level [55]. For this, each nucleoid was classified into one of five classes according to the proportion of DNA in the comet’s tail and head: 0 (no damage); 1 (little apparent damage); 2 (medium damage); 3 (medium damage with longer tail); and 4 (maximum damage). The Damage Index (DI) was thus calculated according to the formula

$$DI=0\times \left(nºclass\;comets\;0\right)+1\times \left(nºclass\;comets\;1\right)+2\times \left(nºclass\;comets\;2\right)+3\times \left(nºclass\;comets\;3\right)+4\times (nºclass\;comets\;4)$$

Thus, the values obtained could range from 0 (no damage) to 400 (maximum damage). Additionally, the Frequency of damage (FD) was calculated based on the percentage of all nucleoids with some damage (class 1 to class 4) relative to the total number of nucleoids counted per animal [56]:

$$FD=\frac{\left(nºofdamaged\;comets-nºclass\;comets\;0\right)\times 100}{\left(nºtotal\right)}$$

All analyses were carried out using a fluorescence microscope (Zeiss Axio Imager. M2, Carl Zeiss AG™, Wetzlar, Germany), with a 40× objective and Alexa Fluor 488 filter (Carl Zeiss AG™, Germany).

#### 4.10.3. Micronucleus Assay

The same mice from the comet test (females *n* = 40) were used, equally with the 8 experimental groups mentioned above. The animals’ blood was collected 48 h after administration. Initially, slides were prepared to receive the biological material, which were washed with neutral detergent, deionized water, bathed in 70% alcohol and placed in an oven at 80 °C for 15 min. Subsequently, 10 µL of acridine orange (1 mg/mL) was spread on the surface of the still heated slides, which were allowed to dry at room temperature for at least 30 min before adding the biological material [57]. Samples of 5 µL of blood from each animal were placed on slides with acridine orange, and a coverslip was positioned over the material [58]. Two slides were prepared per animal, in which 2000 polychromatic erythrocytes (PCE) were analyzed for the presence of micronuclei. The analysis was carried out using a Zeiss Axio Imager M2 fluorescence microscope, with a 40× objective and Alexa Fluor 488 filter (Carl Zeiss AG™, Germany).

### 4.11. Statistical Analysis

To analyze the results of the apoptosis/necrosis assay, the treated groups were compared with the negative control using an ANOVA test, followed by Dunnett’s post-test. In this test, the GraphPad Prisma 8.0 software (http://www.graphpad.com, accessed on 1 May 2024) was used. For the comet test and micronucleus assay, a post hoc analysis was performed using the paired *t* test with Bonferroni correction to compare all groups with each other. The data were analyzed using *R* Software 3.4 (https://www.r-project.org/, was accessed on 1 May 2024). A Shapiro–Wilk test was employed to assess the normality of the data. Data were considered significant at a *p* value < 0.05.

## 5. Conclusions

In summary, our results suggest that BV3 and BV5 derivatives act as promising antineoplastic agents, especially BV3. This study contributes to the understanding of the mechanism of action characteristics in adherent tumor cells, as well as the toxicogenetic safety of these compounds. The findings indicated that BV3 and BV5 may act as antiproliferative agents in HeLa cells by activating apoptosis/necrosis pathways. These compounds showed capacity to inhibit kinases, specially JAK3 and GSK3β. Furthermore, BV3 and BV5 exhibited no mutagenic potential at the tested concentrations, but BV5 at high dose could behave as a possible genotoxic agent. These findings show the potential of BV3 and BV5 in adenocarcinoma of the cervix cells, and critical insights into their mechanisms of action, selectivity, and safety of these compounds.

## Figures and Tables

**Table 1:** Results obtained for the kinase inhibition assay on CDK5/p25, CDK9/CyclinT, HASPIN, PIM1, GSK3β, CK1ε, ABL1, and JAK3. The compounds were tested in duplicate using 10 µM ATP.

	Compounds and Kinases’ Inhibition (% of Residual Activity)					
	BV3		BV5		β-lap	
Kinase	1 µM	10 µM	1 µM	10 µM	1 µM	10 µM
CDK5/p25	≥100	44	≥100	62	74	88
CDK9/CyclinT	≥100	67	≥100	78	≥100	72
HASPIN	≥100	≥100	≥100	≥100	≥100	91
PIM1	≥100	18	94	42	56	42
GSK3β	58	7	64	24	67	43
CK1ε	≥100	98	98	86	≥100	94
ABL1	52	35	83	56	83	81
JAK3	−3	−9	64	4	65	69

**Table 2:** Free binding energies (kcla/mol) and molecular interactions of BV3, BV5, β-lapachone, ML1, and inhibitor 679. (*) ML1 for JAK3 (3XLK) and 679 for GSK3β (1Q4L).

Compound	JAK3 (3LXK)		GSK3β (1Q4L)	
	Free Binding Energy (kcal/mol)	Amino Acids Interactions	Free Binding Energy (kcal/mol)	Amino Acids Interactions
BV3	−10.25	Leu828, Gly829, Lys830, Gly831, Asn832 Gly834, Val836, Ala853, Val884, Met902, Arg953, Leu956, Ala966, Asp967 and Gly969	−9.40	Ile62, Gly63, Asn64, Val70, Ala83, Lys85, Glu97, Val110, Leu132, Asp133, Thr134, Val135, Pro136, Thr138, Arg141, Gln185, Leu188, Cys199, Asp200 and Phe201
BV5	−10.08	Leu828, Gly829, Lys830, Gly831, Val836, Ala853, Val884, Met902, Arg911, Arg953, Leu956 and Ala966	−9.48	Ile62, Val70, Ala83, Lys85, Met101, Leu132, Thr134, Gln185, Leu188, Cys199 and Asp200
β-Lapachone	−8.21	Leu828, Val836, Ala853, Leu905, Leu956 and Ala966	−7.32	Val70, Ala83, Val110, Leu132, Leu188, Cys199 and Asp200
Co-crystallized ligand *	−8.99	Leu828, Ala853, Val884, Met902, Leu905, Cys909, Arg953, Asn954, Leu956 and Ala966	−9.94	Ile62, Val70, Ala83, Lys85, Val110, Leu132, Val135, Arg141, Gln185, Leu188 and Cys199

## Data Availability

All data are contained within the article. Any further information can be provided by the authors upon request.
